# An Offensive Clause

**Published:** 1899-06

**Authors:** 


AN OFFENSIVE CLAUSE.—The general appropriation bill passed by the Twenty-sixth Legislature contains the following clause. We learn that this clause is the principal cause of the opposition with which the bill met on its last round, and that there is much indignation in certain quarters in consequence of its passage. It is feared that it will cause the resignation of some of the asylum superintendents. It is a shame to invite distinguished medical gentlemen to accept the positions of Superintendents and then have the Legislature treat them like they were a lot of thieves. I dare say no other State in the world requires its asylum officers and employes to pay board. It is said that the idea originated with the Governor, and that he recommended that the superintendents be required to board themselves and their families; but we are reliably informed that he recommended also that the Superintendent's salary be raised to $4000. A superintendent remarked to the writer since the passage of this act that his salary is now worth about eight hundred dollars a year. The following is the clause (in part) referred to; it will work a great hardship on the employes, if they have to pay board out of their meagre salaries:

"Sec. 2. * * * * It shall also be unlawful for such superintendent, manager or head of any such institutions or departments to appropriate to his own use, or to the use and benefit of him or his family any of the supplies or material purchased or furnished such institutions or departments under the provisions of this act, or to permit the same to be so used by any of the employes of such institutions or departments, except the salaries and other sums and materials herein expressly granted; provided, that in cases where it is necessary that a teacher or attendant shall be required in any such institution to remain with the inmates of the same, such management may provide for the board and lodging of such teacher or attendant in such institutions, together with such other employes, whose wages are fixed in such manner as makes their board in such institution a part of their compensation. It is made the duty of all such superintendents, managers or heads of such institutions or departments to make report in writing to the Comptroller of this State at least once every three months, and that on the first day of the month, showing the detailed expenditures incurred for the boarding provided for such officers and employes who board and lodge in their respective institutions that may have been drawn and used by the same for the three months next preceding the date of such report. And should it appear from any such reports or from any report of the State Revenue Agent, or *come to the knowledge of such Comptroller from any other reliable source* that any of the provisions of this section have not been complied with, or that the funds of the State or supplies or materials belonging to such institution or department have been misapplied by such superintendent, manager or head of department, or used or permitted to be used as herein prohibited, the Comptroller *shall refuse payment of the salary of such superintendent,* manager or head of department until he shall compensate the State of Texas for the money, supplies or materials that he may have misappropriated, used or knowingly permitted to have been misappropriated or used." [Italics ours.—Ed.]

